# Serial multiple mediation of perceived professional healthcare support and social structural factors in the relationship between care-seeking behavior and perinatal mental health in Chinese mothers

**DOI:** 10.1186/s12889-023-17310-2

**Published:** 2023-12-01

**Authors:** Shanshan An, Sheng Sun

**Affiliations:** Department of Social Medicine, Jiangnan University, Wuxi, 214122 China

**Keywords:** Maternal health, Care-seeking behavior, Healthcare support, Stigma, Trust

## Abstract

**Background:**

In previous studies, it has been observed that mother’s perinatal mental health (PMH) problems can be improved by engaging in appropriate health care-seeking behaviors. However, the exact mechanism that influences the relationship between these two factors is still not fully understood. This cross-sectional study aims to examine the serial multiple mediating effects of perceived professional healthcare support (PPHS) and social structural factors on the association between care-seeking behavior and PMH.

**Methods:**

The cross-sectional study evaluated 1705 Chinese mothers (pregnancy 12 to 41 weeks) through questionnaires from October 2021 to November 2022. These mothers were selected from three hospitals in Wuxi, with an annual delivery volume of at least 5000. We conducted a structural equation model (SEM) analysis to examine the multiple mediating effect of PPHS and social structural factors (social stigma or social trust) between care-seeking behavior and PMH. After analyzing the results of the SEM, we used bootstrapping to further test the mediating effect.

**Results:**

Among the 1705 Chinese mothers surveyed, 636 (37.3%) sought help from professionals. It was observed that more women tended to seek professional help during the early stages of pregnancy (12 to 28 weeks) compared to the later stages (28 to 41 weeks) (t = 1.47, *p* < 0.05). The results of the SEM analysis indicated that the mother’s care-seeking behavior did not have a significant direct effect on PMH. However, it was identified as a crucial distal variable, with its significant effect being fully mediated by PPHS and social structural factors. The mediation effect of PPHS and social stigma on the pathway from care-seeking behavior to PMH was found to be 92.9% (direct effect = 0.002; indirect effect = 0.026). Additionally, the mediating effect of stigma contributed to 21.9% of the association between care-seeking behavior and PPHS (direct effect = 0.405; indirect effect = 0.114). Similarly, the mediation effect of PPHS and social trust on the pathway from care-seeking behavior to PMH was 73.3% (direct effect = 0.008; indirect effect = 0.022). Moreover, the mediating effect of social trust contributed to 22.0% of the association between care-seeking behavior and PPHS (direct effect = 0.407; indirect effect = 0.115). The proposed model showed a good fit with the collected data.

**Conclusion:**

This study examines the serial multiple mediation effect, in which PPHS and social structural factors mediate the relationship between PMH and professional care-seeking behavior. We suggest three levels of intervention: implementing mental health in all policies, providing training for healthcare providers, and establishing healthcare channels with easily accessible information.

## Introduction

Maternal health refers to the health of mother during pregnancy, childbirth and the postnatal period [1]. A previous study showed that by the end of 2021, the maternal mortality rate dropped to 16.1/100,000 in China, far below the median level of middle- and high-income countries (43/100,000) [2]. In contrast, the mental health problems of Chinese mothers are increasing year by year, and 50–75% of primiparas have negative emotions between the 3rd month of pregnancy and the 6th month after birth [3]. The findings suggest a significant rise in anxiety and depressive symptoms among Chinese pregnant and puerperal individuals during the COVID-19 pandemic, who constitute a vulnerable group with respect to mental health morbidity [4]. The Millennium Development Goals relate to the health of mothers and children, which cannot be attained without a specific focus on mother’s mental health [5]. Previous studies have focused on postnatal mental health, particularly postnatal depression, while few studies have been conducted on prenatal mental problems [6]. During the last 10 years, researchers have shown that the incidence of prenatal mental problems is much higher than that of postpartum problems in both high-income countries (HICs) and LMICs [7], and that PMH not only is associated with adverse obstetric and pregnancy outcomes [8], but also has enduring effects on children’s growth and family’s development [9].

When facing mental confusion, a significant number of Chinese pregnant women initially opt for self-regulation and seek informal social support [10]. Recent studies indicate a growing trend among young mothers who prefer seeking assistance from professionals, such as obstetricians and community general practitioners by any action. These professionals provide various services including mental health screening during pregnancy and childbirth, mental health care services throughout the pregnancy and childbirth period, as well as guidance on preventing and treating postpartum depression [11, 12]. The importance of mental health care behavior during pregnancy has been increasingly recognized. It plays a crucial role in identifying and addressing depression in pregnant women, enhancing their awareness of mental health, and empowering them to take better care of their own mental well-being [13, 14]. This, in turn, contributes to the overall health and development of both the mother and the fetus, and facilitates a smoother natural childbirth process [15, 16]. However, the research points out even when seeking professional help, general treatment may not always be enough to provide appropriate mental health care [17]. On the one hand, positive mental health is influenced by variables related to the doctor-patient relationship during the care-seeking process, including the perceptions of mothers regarding the provision of mental health care by professionals [18]. On the other hand, it is subject to many social structural variables, such as stigma and trust [10, 19]. The strong connection between care-seeking behavior and PMH, PPHS, and social factors suggests that these factors may play a crucial role in mediating the relationship between care-seeking behavior and PMH. If this is the case, it could help to bridge the causal gap between seeking professional help and PMH.

As regards the impact of doctor-patient relationship variables, perceived professional healthcare support (PPHS) has a significant impact on PMH. These supports encompass active emotional communication [20], individualized care and treatment [21], continuous provision of care services, and accurate health information [22]. Health professionals have a crucial role in supporting and empowering mothers to cope, develop confidence, and thrive. This can be achieved by adhering to principles such as continuity of care, woman-centered care, informed choice, advocacy, and clinical care [23]. Conversely, when health professionals fail to provide the emotional and affirmational support that mothers need during the immediate postpartum period, their interactions with health professionals may become an additional source of stress instead of alleviating it [24]. Verbal violence or stigmatization of the disease by professionals can also exacerbate psychological unhealth [25]. Chinese women face a major obstacle in the somatization of psychological problems, which often makes it challenging for them to openly share their psychological feelings [26]. This feeling is further reinforced by the lack of recognition and effective practical support from health professionals [27].

As regards the impact of social structural variables such as stigma and trust have significant impact. Stigma refers to the extent to which individuals are concerned about their significant others’ opinion concerning their mental health problems and help-seeking behaviors [28]. Mental health issues are high stigmatized and result in somatization in China [26]. Despite the introduction of policies that include mental health in medical insurance and increased knowledge and understanding of mental health, pregnant women still struggle to cope with daily discrimination related to psychological problems. Several studies have suggested that the stigma associated with perinatal mental health (PMH) can have a negative impact on mothers with mental health problems, leading to a deterioration in their mental health [29, 30]. This stigma not only hinders a mother’s recognition of perinatal distress symptoms [31], but it also influences how young mothers perceive reactions to disclosing their health status to healthcare providers [32]. As another social structural, trust is an important driving force for human relationships of all kinds, and it is a necessity for healthy doctor‒patient relationships. Studies have shown that trust in doctors is associated with positive health behaviors, patient experiences and health outcomes [33]. However, the professional trust of doctors in China is based mainly on the public recognition of the social system [34]. Social trust not only depends on credibility at the hospital system level and the individual level of medical staff but also relies on the establishment of mutually reinforcing expectations through conventions, culture, and upheld promises in the macroenvironment, industry environment and organizational environment [35]. It is important to note that social trust has a significant substitution effect on this influence [34].

Previous studies have shown that structural factor not only affects attitudes towards seeking medical treatment and generating practical behavior, but also influences patients’ perception of professional support [36, 37]. This includes feeling ashamed to reveal one’s inner self and facing difficulty in obtaining care and support that meets their needs, and experiencing fear of stigma and inability to access accurate care information [37]; higher trust can significantly improve the level of mental health of individuals, and can support reliable interactions in unfamiliar social situations, reduce transaction costs, and alleviate stress associated with suspicion [38]. These findings suggest that structural factors play a mediating role.

Existing studies have already established a strong correlation between care-seeking behavior and PMH. However, there is a dearth of research exploring the influencing factors of current medical seeking behavior on positive (negative) mental health in China, and the underlying mechanism connecting these factors remains unclear. Given the strong correlation and predictive effect observed among these variables, it is imperative to develop a theoretical model that elucidates the impact of care seeking on suicidal ideation. Consequently, drawing on previous research findings, this study aims to investigate a serial multiple mediation models, and proposes four hypotheses.

H1: *Care-seeking behavior from professionals has a positive effect on Chinese mother’s PMH.*

H2: *PPHS and social factors plays mediating roles in the relationship between care-seeking behavior and Chinese mother’s PMH.*

H3: *There is a serial multiple mediation effect of social stigma and PPHS on the association between care-seeking behavior and Chinese mothers’ PMH.*

H4: *There is a serial multiple mediation effect of social trust and PPHS on the association between care-seeking behavior and Chinese mothers’ PMH.*

## Methods

### Sample selection

This study is a cross-sectional survey that used a quota sampling method for pregnant women attending three tertiary hospitals located in Wuxi, Jiangsu Province. The number of births per year of hospital A is around 10,000, that of hospital B is above 5300, and that of hospital C is nearly 5100. We used a cross-quota combining two properties of the surveyed hospitals and the gestational period [39, 40]. With the proportion of pregnancies kept balanced (50/50), the sampling ratio of survey places was set at 10/100. A total of 2030 individuals were sampled, included 1000 participants from hospital A, with 500 mothers in early pregnancy (12 to 28 weeks) and 500 mothers in later pregnancy (more than 28 weeks). 530 participants from hospital B, with 260 mothers in early pregnancy and 270 mothers in later pregnancy. 500 participants from hospital C, with 250 mothers in early pregnancy and 250 mothers in later pregnancy. The participants were between 12 and 41 weeks pregnant, and involved in a questionnaire survey of maternal depression generation trajectory and social support research project between February 2022 and October 2022.

### Participants

Participants were recruited through a combination of poster advertisements displayed in the hospital’s outpatient clinic and verbal recruitment. The research team collected questionnaires from various locations, including the obstetric outpatient waiting area, B-ultrasound waiting area, and fetal heart rate monitoring room. At the beginning of the survey, all participants signed an informed consent form. They were guaranteed anonymity and allowed to discontinue the survey at any time. A total of 1899 participants were recruited in this survey: 1899 were recovered, and 1705 were validly answered, for a valid response rate of 89.8%, Hospital A was 771, Hospital B was 457, Hospital C was 477. This research was carried out in compliance with the Helsinki Declaration and with the approval of an appropriate ethics committee, registration number: JNU20211217IRB01.

## Measurement

### Mother’s PMH

The reformulated version of the Body-Mind-Spirit Well-Being Inventory (BMSWBI) was used to measure the mother’s PMH [41]. There is currently no standardized assessment tool for evaluating the psychological well-being of pregnant women in China [11]. Considering the somatization of mental health issues in the Chinese population, it is important to acknowledge that mental health is a complex system that involves various aspects such as cognitions, emotions, values, morality, beliefs, and their interconnectedness with the body. Therefore, in order to gain a comprehensive understanding of the mental health status of Chinese mothers, the author opted to employ a dynamic and systemic scale that is more suitable for this purpose [42]. The final scale consists of 20 items ranked with a 6-point scale (1 = completely inconsistent, 6 = completely consistent). The items were proven to combine into three subfactors (KMO = 0.90, *p* < 0.000) for the body (α = 0.82), mind (α = 0.81) and spirit (α = 0.78) with good reliability (α = 0.87). According to research, there exists a positive correlation between three important factors: body (daily functioning), mind, and spirit. As the score of these factors decreases, it has been observed that mothers tend to experience higher levels of stress and poorer mental health [41].

### Professional care-seeking behavior

The variable for assessing care-seeking behavior from professional included one item based on those from Shwank et al. [10]. A single item for assessing care-seeking behavior asked whether the mother had engaged in any behaviors to seek help from professionals (1 = yes; 0 = no). Care seeking behavior concerning ‘yes’ was defined as situations when women visited any health facility or institution. Conversely, ‘no’ refers to situations when a woman did not consult any healthcare staff.

### Mother’s PPHS

The updated measure of support from medical staff adapted from Olson et al. [43] was used, with three items to evaluate the preciseness of medical staff support (a = 0.71). The items consisted of included the following: “How exact is the emotional support provided by doctors?”, “How exact is the instrumental support provided by doctors?”, and “How exact is the informational support provided by doctors?” The items were rated on a 6-point Likert scale (1 = not at all, 6 = always). These items were proven to combine as one factor (KMO = 0.75, *p* < 0.000) and to explain 73.17% of the variance.

### Social stigma

The variable for assessing social structural included ten items based on those from Shwank et al. [10]. These items were proven to combine into three factors with good reliability (α = 0.77). One factor, “social stigma”, consisted of the following items: “the public thinks that if I seek help from a medical professional, then I have a mental illness”; “the public thinks that if I seek help from a medical professional, then I’m too vulnerable"’ and “the way the public thinks about mental health leads me to blame myself for my negative emotions”. The items were answered on a 6-point Likert scale (1 = disagree strongly, 6 = agree strongly). These items were proven to combine as one factor (KMO = 0.77, *p* < 0.000) and to explain 74.35% of the variance.

### Social trust

A single item was used to assess social trust based on No. 57 of the World Value Survey-7 (WVS) [44]; it was evaluated on a 6-point scale (1 = Do not trust at all, 6 = Trust completely).

### Data analysis

This study employed SPSS 25.0 and AMOS 25.0 software for statistical analysis. Descriptive and correlation analysis were conducted using SPSS 25.0, while AMOS 25.0 was utilized for Structural Equation Modeling (SEM) to examine the serial multiple mediating models of PPHS and social factors on the association between care-seeking behavior and PMH. Our data analysis was conducted in five steps. (1) Descriptive analysis was used to determine the normality of the main variables and obtain the demographic and sociological characteristics of the respondents. (2) Exploratory factor analysis and confirmatory factor analysis were used to determine the structural validity of the data. (3) Reliability checks were performed through reliability analysis. Correlation analysis was used to confirm the relationship between key variables. (4) We employed Structural Equation Modeling (SEM) to assess the predictive ability of care-seeking behavior on PMH. SEM was chosen as a more appropriate method for our study compared to correlation or multiple regression analyses. This is because SEM allows for testing overall models rather than individual coefficients and incorporates multiple dependent and mediating variables [45]. Our analysis focused on two main aspects. Firstly, we examined the parallel mediating effects of PPHS and social factors (stigma or trust) in the relationship between individuals seeking professional care and PMH. Secondly, we verified the mediating effect of social stigma or trust on the relationship between individuals seeking professional care and PPHS, taking into account the influence of structural factors on PPHS. To evaluate the model fit, we utilized established statistics commonly used in SEM [46]: the model fit was assessed based on the chi-square (χ2) test, degrees of freedom, root mean square error of approximation (RMSEA) (recommended ≤ 0.08), comparative fit index (CFI) (recommended > 0.90), Tucker‒Lewis index (TLI) (recommended > 0.90), and normed fit index (NFI) (recommended > 0.9). (5) The bootstrap method was used to calculate the confidence interval of the serial multiple mediating effect and test its significance.

## Results

### Descriptive statistics

Among the 1705 participants (Mean age = 29.57, SD age = 3.70, Max age = 43, Min age = 16), 1000 (58.7%) were giving birth for the first time, 487 (28.6%) for the second time, and 218 (12.7%) for the third time. Regarding household registration certificates, 975 (57.2%) women had Wuxi local household registration, and 730 (42.8%) had nonlocal household registration (refer to Table 1 for more information on demographics).

**Table 1:** Description of the participants’ demographics

Demographics	*N*	*Percentage* (%)
**Household registration certificates**		
Local	975	57.2
Nonlocal	730	42.8
**Number of deliveries**		
One	1000	58.7
Two	487	28.6
Three	218	12.7
**Education**		
High school and below	315	18.5
Bachelor’s degree	1231	72.3
Masters’ degree and above	157	9.2
**Occupation**		
Public institution	396	23.2
Private/foreign-invested enterprise	809	47.6
Freelance	320	18.8
Unemployed	177	10.4
**Monthly household income**		
≤ 10,000	522	30.6
10,001–15,000	482	28.3
15,001–20,000	358	21.0
≥ 20,000	343	20.1

The mean, standard deviation, and correlations of the key variables are shown in Table 2. In terms of the mother’s PMH, the overall score was 4.23, indicating their relatively good mental health. The average score of physical health was the lowest (M = 4.07, SD = 1.11), spiritual health was in the middle (M = 4.12, SD = 0.82), and emotional health was the highest (M = 4.51, SD = 0.99).

Regarding care-seeking behavior, among the 1705 mother, 636 (37.3%) sought help from medical staff. We conducted a T-test to compare the mean values of help-seeking from professionals in early pregnancy and late pregnancy. The results showed that the average score of care-seeking behavior during early pregnancy was the highest (M = 0.40, SD = 0.49), while it was the lowest during the later stages of pregnancy (M = 0.36, SD = 0.48), also found that help-seeking during early pregnancy is easier compared to later stages of pregnancy (t = 1.47, *p* < 0.05). The percentage of Chinese mothers seeking help from professionals is lower compared to informal groups, such as parents (94.6%), friends or colleagues (85.4%), and peers of pregnant mothers (52.5%).

In terms of the mother’s PPHS, the overall score for perception of PPHS was low (M = 1.85, SD = 0.79), with the score for instrumental support being the lowest (M = 2.09, SD = 1.34), followed by emotional support (M = 3.13, SD = 1.45) and informational support (M = 4.05, SD = 1.36). The mean score for social trust was relatively high (M = 4.41, SD = 1.21), and the mean score for social stigma was lower (M = 2.97, SD = 1.22).

**Table 2:** Means, standard deviations, and correlations of the key variables

	M	SD	Physical health		Mental health		Spiritual health		Care-seeking		Emotional		Information		Instrumental		Social stigma	
Body health	4.07	1.11	1															
Mind health	4.51	0.99	0.585	***	1													
Spirit health	4.12	0.82	0.234	***	0.306	***	1											
Care-seeking	-	-	0.048	*	0.026		0.081	**	1									
Emotional	3.12	1.45	-0.005		0.062	*	0.152	***	0.262	***	1							
Informational	4.05	1.36	0.026		0.101	***	0.218	***	0.175	***	0.515	***	1					
Instrumental	2.09	1.35	0.02		0.037		0.116	***	0.243	***	0.473	***	0.35	***	1			
Social stigma	2.97	1.22	-0.214	***	-0.274	***	-0.127	***	-0.051	*	-0.069	***	-0.017		0.003		1	
Social trust	4.41	1.21	0.157	***	0.212	***	0.231	***	0.055	*	0.114	***	0.14	***	0.097	***	-0.059	*

### Correlation analysis between the main variables

As presented in Table 2, there was a positive association between care-seeking behavior and body, mind, and spirit health, indicated by Pearson’s r values of 4.8%, 2.6%, and 8.1%, respectively. Similarly, the three forms of PPHS (emotional, informational, and instrumental) were positively associated with care-seeking behavior, with Pearson’s r values of 26.2%, 17.5%, and 24.3%, respectively. The three forms of professional support and mental health were positively associated. Moreover, social stigma, social trust and mental health were associated with one another. The stronger individuals’ care-seeking behaviors and social stigma in daily life were, the unhealthier they were in terms of mind, body, and spirit, and the poorer their perceived mental health. Conversely, higher scores in PPHS and social trust corresponded to higher levels of mental health and fewer negative moods. Interestingly, in terms of the effect size, the correlation between social factors such as stigma (z1=-21.4%, z2=-27.4%, z3=-12.7%, *p < 0.001*) or trust (z1 = 15.7%, z2 = 21.2%, z3 = 23.1%, *p < 0.001*) and mental health was significantly stronger than the correlation between individual factors and mental health.

### Mediation analysis with structural equation modeling

The structural model was used to test the multiple mediating effect of PPHS and social structural between care-seeking behavior and PMH, and the results are shown in Table 3. Both two measurement models fit the data moderately well, which the fit indices of model A (stigma as a structural factor) showed that χ2 = 117.053, df = 16, CFI = 0.96, TLI = 0.92, RMSEA = 0.060, and model B (trust as a structural factor) were χ2 = 139.22, df = 16, CFI = 0.95, TLI = 0.90, RMSEA = 0.068. The results of the initial model indicated that although care-seeking behavior led to positive PMH, the association was not significant in either Model A (C.R.=0.066, *p = not significant*) or Model B (C.R.=0.418, *p = not significant*), providing partial support for H1.

**Table 3:** Multiple mediation model A and model B

Paths	Estimate		S.E.	C.R.
	B	β		
**Model A (stigma as a social factor)**				
Care-seeking behavior → Health support	0.519	0.326	0.049	10.799^***^
Care-seeking behavior → Social stigma	-0.119	-0.096	0.052	-2.288^**^
Care-seeking behavior → Mental health	0.001	0.002	0.017	0.066
Health support → Mental health	0.031	0.083	0.012	2.527^*^
Social stigma → Health support	-0.040	-0.054	0.020	-2.004^*^
Social stigma → Mental health	-0.084	-0.299	0.010	-8.509^***^
**Model B (trust as a social factor)**				
Care-seeking behavior → Health support	0.522	0.322	0.048	10.793^***^
Care-seeking behavior → Social trust	0.147	0.104	0.061	2.427^**^
Care-seeking behavior → Mental health	0.007	0.012	0.018	0.418
Health support → Mental health	0.024	0.064	0.013	1.883^*^
Social trust → Health support	0.093	0.143	0.018	5.258^***^
Social trust → Mental health	0.062	0.250	0.008	7.574^***^

Model A: χ²=117.053, df = 16, CFI = 0.955, TLI = 0.922, RMSEA = 0.061. *p < 0.05, **p < 0.01, ***p < 0.001

Model B: χ²=139.221, df = 16, CFI = 0.952, TLI = 0.901, RMSEA = 0.068. *p < 0.05, **p < 0.01, ***p < 0.001

As shown in Table 3, the results indicate that both Model A and Model B demonstrate the potential role of PPHS as a mediator in the relationship between care-seeking behavior and PMH (β = 0.326, *p* < 0.001; β = 0.322, *p* < 0.001). Additionally, stigma and trust may also act as another mediators (β=- 0.096, *p* < 0.05; β = 0.104, *p* < 0.05). All of these findings were found to be statistically significant. The study concluded that PPHS played a mediating role in the relationship between care-seeking behavior and the mother’s PMH, while social factors (stigma and trust) played another mediating roles in the relationship between care-seeking behavior and the mother’s PMH. Therefore, H2 was supported.

**Fig. 1:**
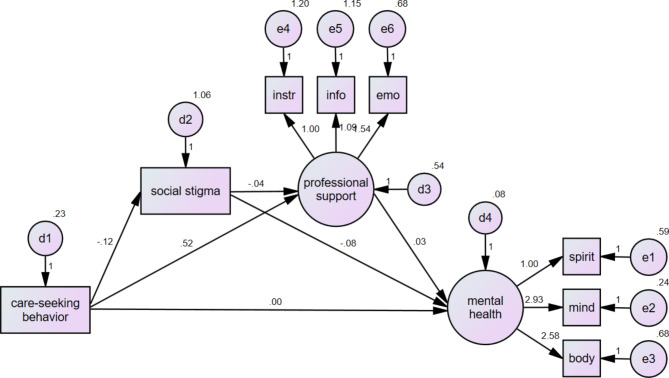
Path analysis of care-seeking behavior, PMH, PPHS, and social stigma

Based on the results of the previous analysis, we utilized bootstrapping to further examine the mediating effect of Model A and Model B. The bootstrap method was employed to estimate the standard errors of the direct and indirect effects of the mediating variables and to present the confidence interval values. The results presented in Table 4; Fig. 1 demonstrate a significant total effect (Std. estimate = 0.028) of professional care-seeking behavior on PMH. Specifically, the indirect effects of care-seeking behavior on PMH through PPHS (Std. estimate = 0.026, *p* < 0.001), and through stigma then PPHS (Std. estimate = 0.114, *p* < 0.001), were found to be significant. Overall, the mediating effect of PPMH and stigma accounted for 92.9% of the pathway from professional care-seeking behavior to PMH. Furthermore, the mediating effect of stigma contributed to 21.9% of the association between care-seeking behavior and PPHS.

**Table 4:** Test of the mediating effect by the bootstrap method

	Total effect	Direct effect	Indirect effect	CI
**Model A (stigma as a social factor)**				
Care-seeking behavior → Health support	0.519	0.405	0.114	0.433-0.650
Care-seeking behavior → Social stigma	-0.119	-0.119	0.000	− 0.209–0.012
Care-seeking behavior → Mental health	0.028	0.002	0.026	− 0.002-0.067
Health support → Mental health	0.031	0.031	0.000	0.008-0.064
Social stigma → Health support	-0.040	-0.040	0.000	− 0.077–0.005
Social stigma → Mental health	-0.085	-0.084	-0.001	.-0.011–0.064
**Model B (trust as a social factor)**				
Care-seeking behavior → Health support	0.522	0.407	0.115	0.436-0.654
Care-seeking behavior → Social trust	0.147	0.147	0.000	0.280-0.350
Care-seeking behavior → Mental health	0.030	0.008	0.022	− 0.006-0.067
Health support → Mental health	0.024	0.024	0.000	0.002-0.055
Social trust → Health support	0.093	0.093	0.000	0.048-0.131
Social trust → Mental health	0.065	0.062	0.002	0.040-0.081

Note: *N* = 1705. Standardized estimates are shown.

**Fig. 2:**
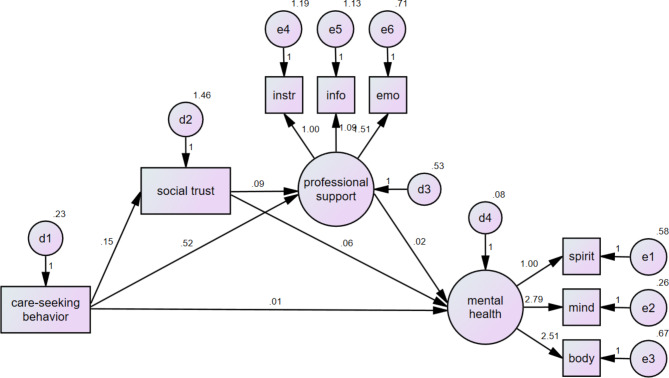
Path analysis of care-seeking behavior, PMH, PPHS, and social trust

Similarly, as shown in Table 4; Fig. 2, there was a significant total effect (Std. estimate = 0.030) of professional care-seeking behavior on PMH. The indirect effects of care-seeking behavior on PMH through PPHS (Std. estimate = 0.022, *p* < 0.001), and through trust then PPHS (Std. estimate = 0.115, *p* < 0.001), were also found to be significant. In this case, the mediating effect of PPHS and social trust accounted for 73.3% of the pathway from professional care-seeking behavior to PMH. Additionally, the mediating effect of trust contributed to 22.0% of the association between care-seeking behavior and PPHS. Therefore, H3 and H4 were supported. Namely, in the context of low levels of social stigma, the positive effect of the path “care-seeking behavior → social stigma → PPHS → mental health” is strongest (H3). In the context of high levels of social trust, the positive effect of the path “care-seeking behavior → social trust → PPHS → mental health” is strongest (H4).

## Discussion

This study is the first to investigate the relationships between individual professional care-seeking behavior, PMH, PPHS, and social structural factors in Chinese women. Our findings reveal a sequential multiple mediation effect, where PPHS and social structural factors mediate the relationship between individual professional care-seeking behavior and individual PMH. Initially, social stigma or trust partially mediated the association between care-seeking behavior and PPHS, followed by PPHS fully mediating the pathway from care-seeking behavior to PMH.

In China, the experience of pregnancy as portrayed by society being natural, satisfying, and joyful. The stigma of being perceived as a ‘failed’ mother could discourage mother from disclosing mental health problems. This study shows that despite negative social perceptions of pregnant women seeking professionals for mental problems, a third of Chinses mothers actively seek help from professionals, and the results align with previous research [11, 12], indicating that an increasing number of young mothers are acknowledging the importance and necessity of seeking professional assistance for their well-being. However, stigma or trust may hinder relationship between pregnant mother and professionals, thus negatively affecting their mental health. This could be caused by the professionals hold more negative views than do other people about outcomes for people with depression during pregnancy [47], due to the fear of being judged or experiencing negative consequences, mothers may refrain from fully disclosing their true condition [48]. That is, professionals’ consultation and treatment that focuses on mental health contributes to stigmatization, whether consciously or unconsciously [49]. Thus, professional reactions in consultations are a significant barrier to disclosure of symptoms and access to support for perinatal mental health symptoms in China. These include a lack of interest in understanding an individual’s mental health history [50], a lack of cultural awareness, and a disregard for the 5R of humility (Reflection, Respect, Attention, Relevance, and Resilience) when dealing with mothers of different genders or races [51], and there seems to be a preference for medication over other non-pharmacological complementary therapies, traditional Chinese medicine, or talk therapy [52]. Stigmatization of treatment hinders mother’s positive perception of professional medical support and the building of trust in the medical communities [53]. We need to further consider and possibly address stigmatic attitudes towards mental health among professionals.

In addition to stigma, social trust was promoting mother’s evaluation of professional support, which has a positive impact on mental health during pregnancy during the process of seeking help from professionals. During China’s urbanization transformation, the spread of the social trust crisis, the ills of the reform of the medical system and the shortcomings of the medical model itself have had a continuous negative impact on individual trust, thus, the interweaving of the social trust crisis [54]. Patients have an intuitive perception of the quality of medical services, medical safety, fairness, and the degree of benefit from medical insurance, this perception plays a crucial role in building trust between patients and doctors [55]. However, the existence of a gap and tension between these factors can hinder the positive effects on patient-doctor trust, thus failing to establish reinforcing dynamics [56]. Therefore, public health campaigns and public policies that widely promote and normalize care-seeking behaviors or care-seeking models may regulate public perceptions of social structural surrounding PMH and lead to greater trust in medical staff.

Factors of support from healthcare professionals emerged as a significant theme in the study. The findings underscore the significance of healthcare professionals offering comprehensive support, encompassing both emotional and technical assistance, in addressing the normal health needs of Chinese mothers. It is crucial for professional support to extend beyond the mere provision of health information or treatments, and instead prioritize a medical approach that centers around women and their overall health experience [57]. This can be achieved by combating prejudice and discrimination related to mental health, respecting women’s values and decision-making autonomy, ensuring patients have a thorough understanding of the information shared during consultations, and offering a range of non-pharmaceutical treatments [51, 52, 56, 58]. Moreover, healthcare providers themselves should address their own mental health concerns, as unaddressed issues can hinder their ability to provide adequate mental health care to perinatal women [57].

### Limitations

Our study has several limitations. Firstly, the survey subjects of this cross-sectional study did not include the psychological status of women in the first three months of pregnancy, as Chinese obstetrics departments only accept pregnant women after 12 weeks of pregnancy. The results of another study which involved one-on-one interviews with 20 women in early pregnancy from gynecology, reproductive, and other departments, are not presented in this article. Secondly, the generalizability of the findings is limited due to the restricted geographical diversity in China. Future research will expand the geographic scope of the survey and utilize random sampling methods to mitigate sample bias and enhance diversity. This will enable stronger validation of the generalizability of the findings. Thirdly, while this study considered a two-factor mediation model, it is possible that other mediating or moderating variables exist. Therefore, future research could examine the mental health status of pregnant women at multiple time points throughout pregnancy and delivery, conduct long-term follow-up studies, expand the model to assess the impact on maternal mental health, and provide evidence for improving maternal health.

## Conclusion

This study analyzed the relevant factors affecting mother’s care-seeking behavior translated into positive PMH. These results suggest that even when mother seeks professional medical help for mental health issues, it cannot have a direct impact on her positive mental health. The relationship between the Chinese mother and healthcare professionals can be influenced by factors such as the support provided by medical staff, social stigma, and social trust. In other words, there are structural issues in the process of Chinese mothers seeking medical care. The stigma surrounding psychological problems hinders them from openly discussing their true condition with professionals, while their lack of trust in the medical system prevents them from accepting professional advice. These factors can negatively impact the relationship between Chinese mothers and healthcare providers, ultimately having a detrimental effect on their mental health. Beginning 2022, Jiangsu Province has included psychotherapy projects in the scope of medical insurance payments in China to better meet the psychotherapy needs of patients and reduce the cost of treatment. Chinese policy emphasizes the importance of integrating mental health into prenatal and primary care. However, current approaches have predominantly focused on ‘treatment’ rather than ‘health care’ and have been influenced by personal behavior, consistent relationships, and social structural factors. This narrow focus has resulted in missed opportunities. The findings presented in this study highlight the complex factors that contribute to the healthcare process and ultimately hinder the promotion of mental health among Chinese women during pregnancy.

According to the results of this article, the effectiveness of care-seeking behavior can be improved in three ways. Healthcare providers should consider implementing the following strategies to augment support for women during the perinatal period. The first is to take effective actions at the country level that implement country level actions that integrate mental health into antenatal and perinatal care. Additionally, comprehensive and multi-layered interventions should be established to eradicate stigma and build trust, targeting various risk factors associated with mental health. Another crucial aspect is to improve education and training for mental health professionals. This can help foster positive attitudes towards mental illness and promote the use of non-drug treatment interventions, such as mental health education, therapeutic optimism, and the expansion of support networks. It is also important to connect individuals with formal and informal social services. Furthermore, early intervention is essential, starting from early pregnancy. Women should be provided with accurate and consistent information about mental healthcare, along with accessible care-seeking channels. It is crucial to support women in accessing the right services at the right time.

## Data Availability

The data that support the findings of this study are available on request from the corresponding author. The data are not publicly available due to their containing information that could compromise the privacy of research participants.
